# Supplementary material for: Schistosome eggs stimulate reactive oxygen species production to enhance M2 macrophage differentiation and promote hepatic pathology in schistosomiasis
Source: PLoS Negl Trop Dis. 2021 Aug 16;15(8):e0009696. doi: 10.1371/journal.pntd.0009696 (PMC8389433; doi:10.1371/journal.pntd.0009696)
Supplement: Attachment — Submitted filename: Response to Reviewers.docx [file pntd.0009696.s001.docx]

We truly appreciate to have been given the opportunity to revise our manuscript. We are pleased that the reviewers consider our findings to be interesting and significant. Thank the reviewers for the helpful and constructive comments. We have carried out additional experiments, added 3 new figures (Figure 1A, Figure 1D, and Figure 2F), and extensively revised our manuscript according to the reviewers' comments. The newly acquired results further supported the conclusions described in the manuscript.

Our point-by-point responses to the reviewers’ comments are described below.

Reviewer #1: Interesting study on ROS and hepatic schistosomiasis. Experiments were comprehensive, performed well, and results presented effectively.

Response: We would like to thank the reviewer for considering our study interesting and our data comprehensive and presented effectively.

Figure 1 (and others), All panels need a scale bar. Panels B and C do not need the staining type labelled, this information only needs to be in the legend. While the images of livers were informative the use of weights would have been more precise and could have enabled statistical comparison.

Response: Following the reviewer’s suggestion, we have added scale bars to all the relevant previous figures and the new Figure 1A, and removed the redundant information from Figures 1B&C and Figure 2B&C. We have also revised our manuscript (lines 239 and 595-596 of marked version).

Figure 4 please clarify the four groups especially the difference between “-“ and “saline” groups. Does “-“ indicate no oral dosage at all?

Response: We are sorry for the lack of these details. We have added the information in the legend of Figure 4 in our revised manuscript as following: *S. japonicum*-infected mice were untreated (-) or treated with saline or Apo orally (lines 630-631 of marked version).

Line 66 “(S. mansoni)” is not needed and further species only require the abbreviated genus name ie delete “Schistosoma japonicum”.

Response: We appreciate the reviewer’s suggestion and have modified our manuscript accordingly as following: *Schistosoma mansoni* (*S. mansoni*), *S. japonicum*, and *S. haematobium* are schistosome species of major medical importance (lines 67-68 of marked version).

Line 80 please add reference.

Response: We apologize for missing the reference that has now been added to our revised manuscript (line 81 of marked version).

What lobe of the liver was used for histology?

Response: We are sorry for this missing information that has now been added to our revised manuscript as following: The right lower lobe of the liver was harvested from infected mice and fixed in 4% buffered formalin, embedded in paraffin, and then sectioned at 5 to 7 μm (lines 135-136 of marked version).

Can you confirm that LSP was demonstrated as LPS free?

Response: We are sorry for the lack of these details. We have added the information in the Materials and Methods section of our revised manuscript as following: The supernatant was recovered by centrifugation and filter-sterilized with a 0.22 μm filter and the endotoxin was removed with Polymyxin B-Agarose (Sigma-Aldrich). The remaining endotoxin activity (<0.01 EU/μg) was determined using the LAL assay kit (ThermoFisher Scientific, MA, USA) (lines 161-163 of marked version).

Line 242 (Figure 5C-5E) should be fig 2? Similarly on line 273?

Response: Thank the reviewer for the careful review of our manuscript. We have corrected these mistakes in our revised manuscript (lines 260 and 293 of marked version).

Discussion was well written and was effectively supported by appropriate references. Line 375 it would be nice for the authors to propose future experiments to expand their findings below “….need to be further explored.”.

Response: We thank the reviewer for the suggestion. We have revised the Discussion by adding the following points: Future studies, e.g., deeper investigation of the roles and mechanisms of NADPH oxidase- or mitochondria-derived ROS production in schistosomiasis, should be conducted (lines 398-400 of marked version).

Reviewer #2: This manuscript describes an investigation into the association between ROS production, macrophage differentiation and the development of hepatic pathology during infection with S. japonicum. The major finding from this study is that a significant increase in ROS in the liver of infected mice is causally linked to the formation of granulomas, as the administration of a specific inhibitor of NOX reduced pathology. In addition, the presence of this inhibitor also impacted the developing immune response in animals, reducing the differentiation of M2-type macrophages and increasing the population of M1 cells in the liver, and reducing the number of IL-4 secreting T cells in the spleen. This was partly repeated in vitro, where the presence of the inhibitor prevented the SEA induced differentiation of M2 like cells, but unlike the scenario in vivo, did not enhance the development of M1. The in vitro analysis also attempted to shed some light on the possible mechanism by which the SEA drives ROS-mediated differentiation of macrophages, with data suggesting a Syk-dependent activation of NOX. The outcomes contribute to improving understanding of the progression of liver pathology after infection with Schistosoma and how the parasite may be influencing the development of specific macrophage phenotypes.

The study utilised standard protocols which were performed well and interpreted correctly. Although, it should be clarified in text whether apocynin is a specific inhibitor of NOX-2.

Response: We appreciate to have been given the opportunity to revise our manuscript. Thanks for the suggestion. We have now included this information in the Materials and Methods section of our revised manuscript as following: Infected mice were randomly chosen for treating with an NADPH oxidase 2 (NOX2) inhibitor, apocynin…… (line 130 of marked version).

However, while the data has shown that SEA induces macrophage differentiation at least in part via ROS production and that the ROS in the liver contributes to the pathology, the data does not show (as suggested by the authors) that the macrophages then contribute to the granuloma formation. I would suggest that this conclusion (Line 377) is re-considered.

Response: We fully agree with the reviewer and have revised our manuscript to make the conclusion more appropriate as following: In summary, our study shows that ROS accumulation in the liver of *S. japonicum-infected* mice contributes to hepatic immunopathology and further demonstrates that schistosome eggs induce ROS generation to promote M2 macrophage differentiation (lines 401-405 of marked version). We also have revised the title of our manuscript as following: Schistosome eggs stimulate reactive oxygen species production to enhance M2 macrophage differentiation and promote hepatic pathology in schistosomiasis.

In addition, there is no evidence that the CD206+ macrophages are driving the differentiation of IL-4 secreting T-cells; it may in fact equally be the other way around and the IL-4 produced by T-cells is driving the differentiation of macrophages in vivo. While the data is presented accurately, the conclusions drawn need to be reconsidered – as the experimental design does not support the demonstration of causal sequential cascade of SEA – ROS- M2 - granuloma/Th2.

Response: We thank the reviewer for pointing out this important issue. We have modified the related result section and added some discussion to more precisely reflect the conclusion of our manuscript as following: (1) The revised conclusion of Figure 4: ROS also play an important role in enhancing Th2 responses during *S. japonicum* infection (lines 296-297 of marked version). (2) The revised Discussion part: Considering reciprocal regulation between macrophages and Th cells, ROS-enhanced M2 macrophages and Th2 cells are likely to have a relationship of mutual promotion (lines 368-370 of marked version).

Some minor comments:

1. Introduction Line 78: M2 macrophages are not phenotypically the same as alternatively activated macrophages, which have been characterised specifically as IL-4 activated macs. Please refer to the recent literature around the nomenclature of macrophages

Response: Thanks for the reviewer’s kind reminder. We have removed the nomenclature of “alternatively activated macrophages” in our revised manuscript.

2. Results Line 220: I would suggest replacing “Massive” with more scientific terminology

Response: Thanks for pointing out this misnomer. We have revised our manuscript as following: Increased ROS are accumulated in the livers of *S. japonicum*-infected mice (lines 235 and 246 of marked version).

3. Some of the figure numbers are incorrectly listed throughout the results text

Response: We thank the reviewer for the careful review. We have checked the full text and corrected all the incorrect figure numbers in our revised manuscript (lines 260 and 293 of marked version).

4. Fig 1: scale bars should be added to the histology figures and described in the legend.

Response: Thanks for this suggestion. We have added scale bars to all the relevant figures and revised our manuscript (lines 598 and 609 of marked version).

5. Fig 1: I am not convinced by the DCFH-DA staining which is very diffuse and almost non-specific. It may help the interpretation of this data if the tissue can be counterstained for some other liver/granuloma structure – or even DAPI at the most basic.

Response: Following the reviewer’s suggestion, we have carried out additional experiments and used DAPI for nuclear counterstaining to better visualize the liver/granuloma structure in ROS assay. Consistently, our newly acquired results showed that increased ROS are accumulated in the livers of *S. japonicum*-infected mice. We have added these newly acquired data in Figure 1D and revised our manuscript (lines 154-155 and 598-600 of marked version). We believe it would further strengthen our conclusion.

6. Fig 2: It would be of value here to have a measure of liver health to accompany the assessment of pathology in the liver (perhaps serum ALT or equivalent). While the livers certainly seem smaller in the animals treated with APO, they do not look any healthier.

Response: Following the reviewer’s suggestion, we have carried out additional experiments to further evaluate the liver pathology. Our newly acquired results showed that Apo treatment also significantly reduced the levels of AST and ALT in *S. japonicum*-infected mice. We have added these newly acquired data in Figure 2F and revised our manuscript (lines 176-179, 260-261, and 612-633 of marked version).

7. Line 276; as mentioned the data does not show that macrophage activation is subsequently controlling the Th1/Th2 response; it does show that both CD206+ macrophages and Th2 responses are present in infected animals and reduced in animal given Apo.

Response: We fully agree with the reviewer and have revised our manuscript to make the conclusion more appropriate as follows (line 295 of marked version): ROS also play an important role in enhancing Th2 responses during *S. japonicum* infection.

8. Fig 6: The sample size should be added to the legend for this figure

Response: We are sorry for the lack of this information that has now been added to our revised manuscript as following: Data are representative of two independent experiments, each with triplicates (line 660 of marked version).

9. There are a number of language and typo errors throughout.

Response: Thanks for the reviewer’s kind reminder, and we have carefully checked the manuscript thoroughly and corrected the misspellings and grammatical errors in our revised manuscript.
